# Recovery of right ventricular function after intermediate-risk pulmonary embolism: results from the multicentre Pulmonary Embolism International Trial (PEITHO)-2

**DOI:** 10.1007/s00392-022-02138-4

**Published:** 2022-12-21

**Authors:** Anna C. Mavromanoli, Stefano Barco, Walter Ageno, Hélène Bouvaist, Marianne Brodmann, Claudio Cuccia, Francis Couturaud, Claudia Dellas, Konstantinos Dimopoulos, Daniel Duerschmied, Klaus Empen, Pompilio Faggiano, Emile Ferrari, Nazzareno Galiè, Marcello Galvani, Alexandre Ghuysen, George Giannakoulas, Menno V. Huisman, David Jiménez, Matija Kozak, Irene M. Lang, Nicolas Meneveau, Thomas Münzel, Massimiliano Palazzini, Antoniu Octavian Petris, Giancarlo Piovaccari, Aldo Salvi, Sebastian Schellong, Kai-Helge Schmidt, Franck Verschuren, Irene Schmidtmann, Gerrit Toenges, Frederikus A. Klok, Stavros V. Konstantinides, The PEITHO-2 Investigators, Jaime Antonio Abelaira Freire, Ibrahim Akin, Toni Anusic, Dorothea Becker, Laurent Bertoletti, Giuseppe Bettoni, Harald Binder, Regina Carels, Giuseppe Di Pasquale, Daniel Dürschmied, Iolanda Enea, Joachim Ficker, Sabine Genth-Zotz, Philippe Girard, Stanislav Gorbulev, Matthias Held, Lukas Hobohm, Menno V Huisman, Stavros V Konstantinides, Kai Kronfeld, Irene Marthe Lang, Mareike Lankeit, Walter Lehmacher, Concepcion Patricia Lopez Miguel, Nadine Martin, Guy Meyer, Roman Pareznik, Kurt Quitzau, Irinel Raluca Parepa, Purificacion Ramirez Martin, Marc Righini, Silviu Bogdan Todea, Adam Torbicki, Luca Valerio, Thomas Vanassche, Luminita Animarie Vida-Simiti, Anamaria Wolf-Pütz

**Affiliations:** 1 Center for Thrombosis and Hemostasis, University Medical Center of the Johannes Gutenberg University, Langenbeckstr. 1, 55131 Mainz, Germany; 2 Department of Angiology, University Hospital Zurich, Zurich, Switzerland; 3 Department of Medicine and Surgery, University of Insubria, Varese, Italy; 4 Department of Cardiology, Pôle Thorax et Vaisseaux, CHU Grenoble Alpes, La Tronche, France; 5 Department of Angiology, Medical University Graz, Graz, Austria; 6 Cardiovascular Department, Fondazione Poliambulanza, Istituto Ospedaliero, Brescia, Italy; 7 Département de Médecine Interne et Pneumologie, Centre Hospitalo-Universitaire de Brest, Brest, France; 8 INSERM U1304-GETBO, FCRIN INNOVTE, Brest University, Brest, France; 9 Clinic of Paediatric Cardiology and Intensive Care, ACHD Center, University Medical Center Goettingen, Goettingen, Germany; 10 Royal Brompton Hospital, London, UK; 11 National Heart and Lung Institute, Imperial College, London, UK; 12 Department of Cardiology, Angiology, Haemostaseology and Medical Intensive Care, University Medical Centre Mannheim, Medical Faculty Mannheim, Heidelberg University, Mannheim, Germany; 13 Department of Internal Medicine B, University Medicine Greifswald, Greifswald, Germany; 14 Fondazione Ospedaliera Poliambulanza, Brescia, Italy; 15 Service de Cardiologie, Hôpital Pasteur, Centre Hospitalier Universitaire de Nice, Nice, France; 16 Cardiology Unit, IRCCS Azienda Ospedaliero and Dipartimento DIMES-Università di Bologna, Bologna, Italy; 17 Division of Cardiology, Department of Cardiovascular Diseases - AUSL Romagna, Ospedale Morgagni-Pierantoni, Forli, Italy; 18 Cardiovascular Research Unit, Fondazione Cardiologica Myriam Zito Sacco, Forli, Italy; 19 Emergency Care, University Hospital Centre Liège, Liège, Belgium; 20 Cardiology Department, AHEPA University Hospital, Aristotle University of Thessaloniki, Thessaloniki, Greece; 21 Department of Medicine - Thrombosis and Hemostasis, Leiden University Medical Center, Leiden, The Netherlands; 22 Department of Respiratory Diseases, Ramon y Cajal Hospital, Universidad de Alcalá (IRYCIS), CIBER Enfermedades Respiratorias (CIBERES), Madrid, Spain; 23 Department of Vascular Diseases, University Medical Center, Ljubljana, Slovenia; 24 Department of Cardiology, Medical University of Vienna, Vienna, Austria; 25 Department of Cardiology, University Hospital Jean Minjoz, Besançon, France; 26 EA3920, University of Burgundy Franche-Comté, Besançon, France; 27 Department of Cardiology, University Medical Center of the Johannes Gutenberg University, Mainz, Germany; 28 Cardiology Clinic, “St. Spiridon” County Clinical Emergency Hospital, Grigore T. Popa University of Medicine and Pharmacy Iasi, Iasi, Romania; 29 Department of Cardiovascular Diseases, Infermi Hospital, AUSL Romagna, Rimini, Italy; 30 Internal and Subintensive Medicine Department, Azienda Ospedaliero-Universitaria “Ospedali Riuniti” di Ancona, Ancona, Italy; 31 Department of Internal Medicine 2, Municipal Hospital Dresden, Dresden, Germany; 32 Emergency Department, Cliniques Universitaires Saint-Luc, Université Catholique de Louvain, Brussels, Belgium; 33 Institute of Medical Biostatistics, Epidemiology and Informatics (IMBEI), University Medical Center of the Johannes Gutenberg University, Mainz, Germany; 34 Department of Cardiology, Democritus University of Thrace, Alexandroupolis, Greece

**Keywords:** Echocardiography, Right ventricle, Dysfunction, Pulmonary embolism, Intermediate-risk

## Abstract

**Background:**

Right ventricular (RV) function plays a critical role in the pathophysiology and acute prognosis of pulmonary embolism (PE). We analyzed the temporal changes of RV function in the cohort of a prospective multicentre study investigating if an early switch to oral anticoagulation in patients with intermediate-risk PE is effective and safe.

**Methods:**

Echocardiographic and laboratory examinations were performed at baseline (PE diagnosis), 6 days and 6 months. Echocardiographic parameters were classified into categories representing RV size, RV free wall/tricuspid annulus motion, RV pressure overload and right atrial (RA)/central venous pressure.

**Results:**

RV dysfunction based on any abnormal echocardiographic parameter was present in 84% of patients at baseline. RV dilatation was the most frequently abnormal finding (40.6%), followed by increased RA/central venous pressure (34.6%), RV pressure overload (32.1%), and reduced RV free wall/tricuspid annulus motion (20.9%). As early as day 6, RV size remained normal or improved in 260 patients (64.7%), RV free wall/tricuspid annulus motion in 301 (74.9%), RV pressure overload in 297 (73.9%), and RA/central venous pressure in 254 (63.2%). At day 180, the frequencies slightly increased. The median NT-proBNP level decreased from 1448 pg/ml at baseline to 256.5 on day 6 and 127 on day 180.

**Conclusion:**

In the majority of patients with acute intermediate-risk PE switched early to a direct oral anticoagulant, echocardiographic parameters of RV function normalised within 6 days and remained normal throughout the first 6 months. Almost one in four patients, however, continued to have evidence of RV dysfunction over the long term.

**Graphical Abstract:**

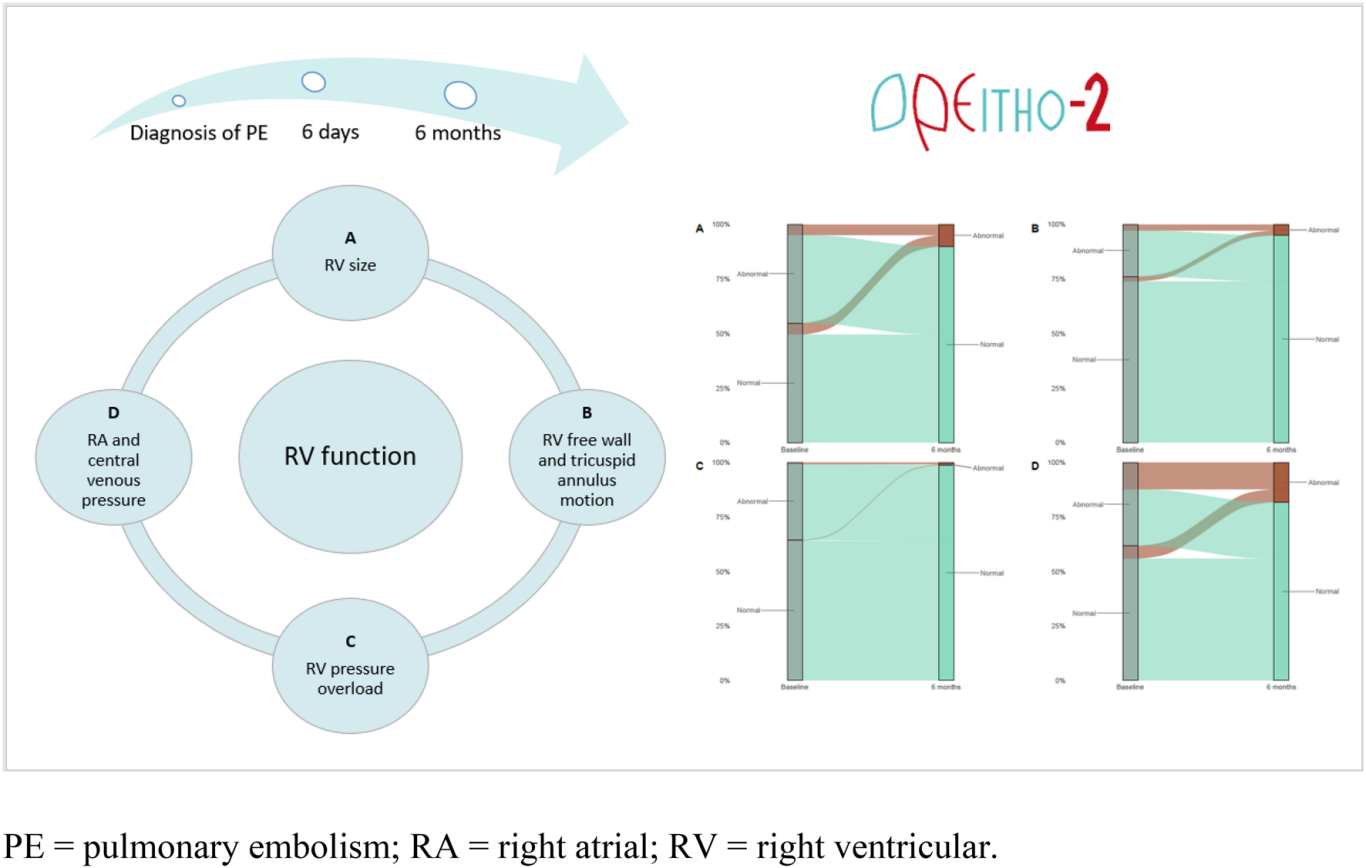

**Supplementary Information:**

The online version contains supplementary material available at 10.1007/s00392-022-02138-4.

## Introduction

Impairment of right ventricular (RV) function resulting from acute RV pressure overload plays a critical role in the pathophysiology and prognosis of pulmonary embolism (PE) [1, 2]. In particular, the combination of RV dilatation with RV ischaemia, injury and inflammation may lead to overt RV failure causing haemodynamic instability and death [3]. RV dysfunction, indicated by abnormal echocardiographic signs or elevated cardiac biomarkers, has been shown to predict short-term mortality in patients with PE even in the absence of clinically evident haemodynamic compromise at presentation [4], while signs of RV dysfunction at discharge have previously been associated with PE-related death [5].

Among survivors of the acute phase of PE, approximately 20% have been reported to present with persistent RV dysfunction at follow-up [5]. In fact, in patients with intermediate-risk PE participating in the Pulmonary Embolism Thrombolysis (PEITHO) trial, absence of complete RV recovery at 6 months, as assessed by echocardiography, predicted persisting RV dysfunction over the entire two-year follow-up period [6]. However, the definition of RV dysfunction has not been standardised, with various echocardiographic parameters having been used over the years, while laboratory values have also been considered in some cohorts [7–9].

The prospective multicentre single-arm Pulmonary Embolism International Trial (PEITHO)-2 (ClinicalTrials.gov Identifier NCT02596555, EudraCT Identifier 2015-001830-12) investigated if the early switch from parenteral heparin to oral anticoagulation using dabigatran in patients with intermediate-risk PE is effective and safe [10]. The present predefined analysis from the PEITHO-2 study sought (a) to determine the temporal pattern of recovery of RV function, as assessed by echocardiographic and biochemical parameters, in the PEITHO-2 study population; and (b) to identify baseline predictors of RV dysfunction during follow-up.

## Methods

The rationale and design of the PEITHO-2 study have been previously described [11]. The main inclusion criteria in the study were an age of at least 18 years and the objective diagnosis of intermediate-risk PE, based on the classification proposed by the 2014 European Society of Cardiology (ESC) guidelines [12]. Key exclusion criteria were: pregnancy, reduced life expectancy, haemodynamic instability at presentation, presence of active bleeding or high risk for bleeding, contraindications to dabigatran, need for long-term anticoagulation/reperfusion treatment and impaired kidney/liver function. The primary efficacy endpoint was recurrent symptomatic venous thromboembolism (VTE) or PE-related death within 6 months after the index PE event.

**Fig. 1:**
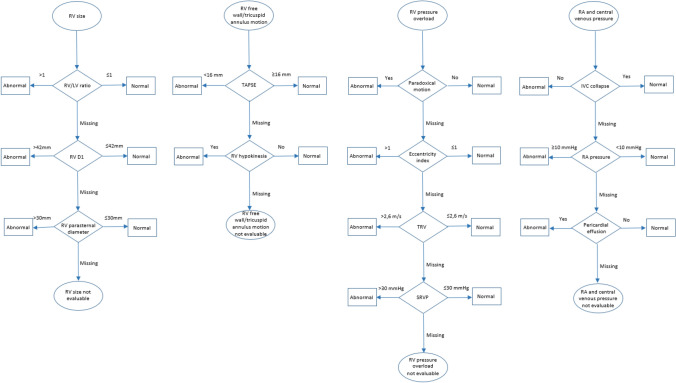
Algorithm for echocardiographic assessment of right ventricular function during follow-up based on four key categories of ultrasound parameters. *D1* basal end-diastolic diameter, *IVC* inferior vena cava, *LV* left ventricular, *RA* right atrial, *RV* right ventricular, *SRVP* systolic right ventricular pressure, *TAPSE* tricuspid annular plane systolic excursion, *TRV* tricuspid regurgitant jet velocity

According to the study protocol, echocardiographic and laboratory examinations were performed at baseline, i.e. upon enrolment, as well as at the 6-day and 6-month follow-up. To permit a standardised, coherent and complete assessment of the echocardiographic follow-up and comparison with the baseline status, all measured echocardiographic parameters (predefined; based on the protocol of the PEITHO-2 study [11]) were *prospectively* classified for the present analysis into four categories or groups, each one corresponding to a key manifestation of RV pressure overload cardiac imaging (Table 1): (i) RV size; (ii) RV free wall and tricuspid annulus motion; (iii) RV pressure overload; and (iv) right atrial (RA) and central venous pressure. This classification was not designed on the assumption that the above groups of findings are pathophysiologically ‘independent’ from each other; instead, it was implemented to ensure complete and reproducible echocardiographic reports in each patient and at each visit, based on the main pathophysiologic mechanisms implicated in RV dysfunction. In that sense, abnormal RV size (dilatation) was primarily confirmed by the documented right-to-left ventricular (RV/LV) end-diastolic diameter ratio; as a second option, if this parameter was missing, by the basal (D1) end-diastolic diameter of the RV measured in the 4-chamber view; and as a third option, if both of the above parameters were not available, by the RV end-diastolic diameter measured in the parasternal view. With a similar rationale, reduced RV free wall and tricuspid annulus motion was primarily evaluated by the tricuspid annular plane systolic excursion (TAPSE); or, if TAPSE was missing, by visual confirmation of RV free wall hypokinesia. RV pressure overload was indicated by visual confirmation of paradoxical septal wall motion; or by an increased LV eccentricity index indicating septal flattening and LV diastolic compression; and, as a third option, by an elevated (estimated) RV systolic pressure documented by measuring the tricuspid regurgitant jet velocity and calculating systolic RV pressure via the Bernoulli equation. Finally, increased RA and central venous pressure were primarily diagnosed by the documented absence of inspiratory collapse of the inferior vena cava and semi-quantitative estimation of right atrial pressure; or by the presence of pericardial effusion not explained by an alternative diagnosis (Fig. 1).

**Table 1:** Echocardiographic criteria for detecting and classifying right ventricular dysfunction at baseline and during follow-up, with corresponding cut-off values

Category	Parameters	View	Cut-off
RV dilatation	Increased RV/LV end-diastolic diameter ratio	Apical or subcostal 4-chamber	> 1.0
	Increased RV D1 end-diastolic diameter	Apical 4-chamber	> 42 mm
	Increased RV end-diastolic diameter	Parasternal long or short axis (papillary muscle level)	> 30 mm
Reduced RV free wall and tricuspid annulus motion	Reduced TAPSE	Apical 4-chamber (modified)	< 16 mm
	RV free wall hypokinesia	Apical 4-chamber	Yes (visually)
RV pressure overload	Paradoxical motion of the interventricular septum	Apical 4-chamber	Yes (visually)
	Septal flattening: LV eccentricity index (diastole)	Parasternal short axis (papillary muscle level)	> 1.0
	Increased tricuspid regurgitant velocity	Apical 4-chamber	> 2.6 m/s
	Increased systolic RV pressure (calculated)	Apical 4-chamber	> 30 mmHg
Increased RA and central venous pressure	Absence of inspiratory collapse of the inferior vena cava	Subcostal	< 50% diameter reduction
	Increased RA pressure (estimated)	Subcostal	≥ 10 mmHg
	Pericardial effusion	Subcostal	Yes (visually)

*D* diameter, *LV* left ventricular, *RA* right atrial, *RV* right ventricular, *TAPSE* tricuspid annular plane systolic excursion

The analysis of echocardiographic data required assessment of each one of the four categories as defined above, both at baseline and during follow-up. The ‘hierarchical’ order applied to define an abnormal status within each category was based on existing evidence and expert consensus on the relative prognostic strength and reproducibility of individual ultrasound parameters [1, 13]. Comparing the echocardiographic parameters at different time points and using the cut-off values shown in Table 1, the course of RV function during follow-up was described as: (a) persistently abnormal; (b) deteriorating; (c) improving; or (d) remaining normal. The laboratory parameter used for the assessment of RV dysfunction was the N-terminal pro-brain natriuretic peptide (NT-proBNP) level. The levels of NT-proBNP were considered abnormal if being above the cut-off value of 125 pg/ml [14, 15].

Statistical analysis was performed in the intention-to-treat population [10]. Categorical variables are reported with absolute and relative frequencies; continuous variables, with the corresponding median and interquartile range. Alluvial plots were designed to depict the course of RV function, as assessed by echocardiography, during follow-up. Univariable and multivariable logistic regression models were applied for examining predictors of abnormal RV function at 6 days and 6 months. The variables included in the models were selected on the basis of existing literature and current medical knowledge [1]. The results are presented as odds ratios with the corresponding 95% confidence intervals. The R software (R: A language and environment for statistical computing. R Foundation for Statistical Computing) was used for the statistical analysis.

## Results

A total of 402 patients with intermediate-risk PE (48% women, median age of 69.5 years; 70% in the intermediate-high risk category) were enrolled in the PEITHO-2 study between January 2016 and July 2019. Patients were followed for 180 days after enrolment. During follow-up, 7 (2%) patients developed recurrent symptomatic VTE or PE-related death, 8 (2%) died from any cause and 11 (3%) had major bleeding [10].

Echocardiographic signs of RV dysfunction based on any abnormal parameter were present in 84% (*n* = 338) of the patients at baseline. RV dilatation was the most frequently abnormal echocardiographic finding (163 patients, 40.6% of the total study population), followed by increased RA and central venous pressure (139 patients, 34.6%), RV pressure overload (129 patients, 32.1%), and reduced RV free wall and tricuspid annulus motion (84 patients, 20.9%; Supplementary Table S1). The baseline characteristics of the patients with complete as opposed to those without completely available echocardiographic data at 180-day follow-up are shown in Table S2. After the acute phase of PE, the frequency of all markers of RV dysfunction decreased substantially; at 6 months, RV dysfunction had recovered in the vast majority of the patients, with RV enlargement and dilated inferior vena cava being the only findings documented somewhat more frequently, in 8.2% and 16.4% of the patients, respectively (Table S1).

**Fig. 2:**
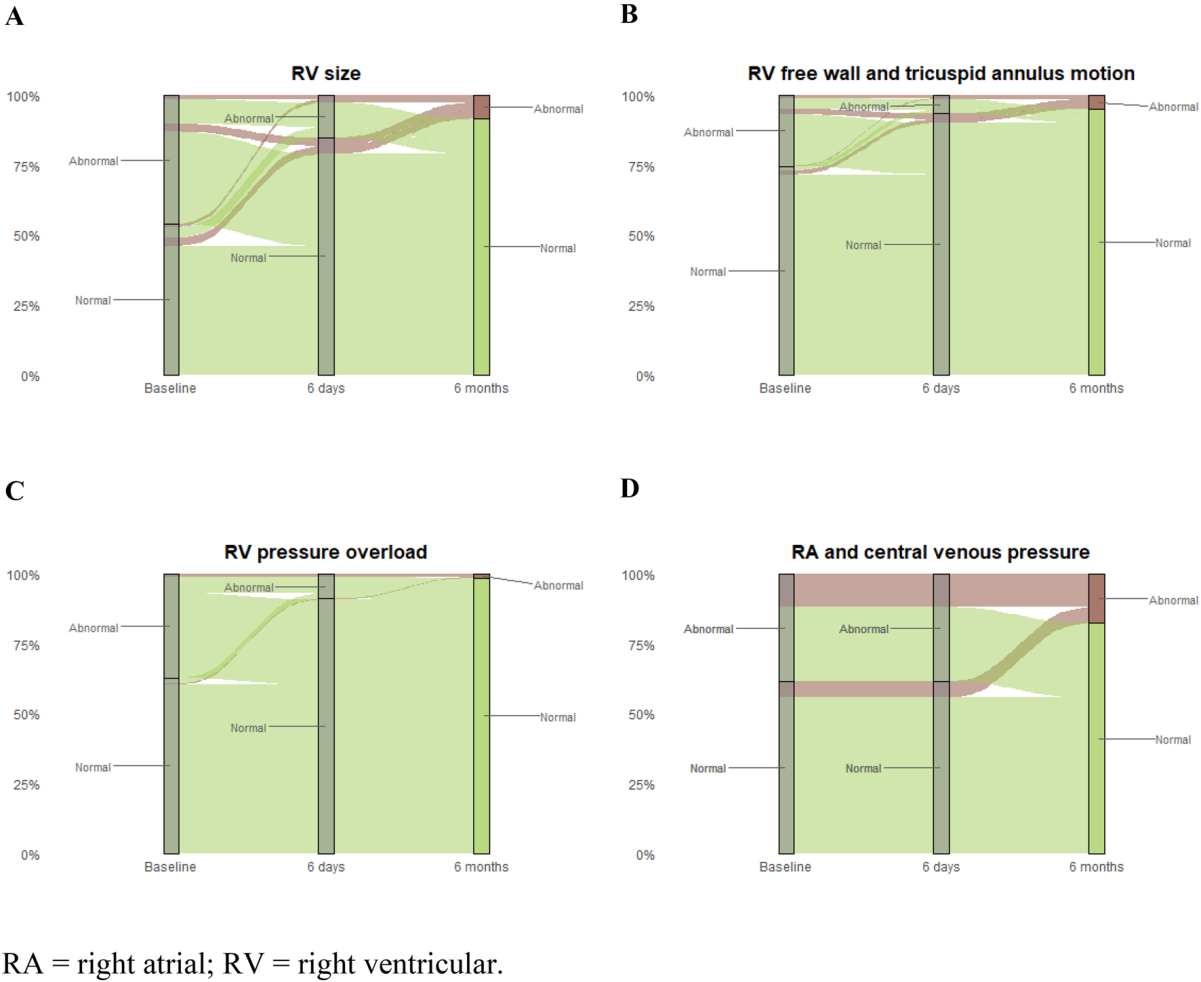
Changes in echocardiographic parameters of right ventricular function across the 6-month follow-up: green color represents ‘normal’ and red ‘abnormal’ result at the end of follow-up. *RA* right atrial, *RV* right ventricular

Figure 2 displays the temporal changes of RV function across visits as assessed by echocardiography. As early as day 6, remaining normal or improved (from baseline) RV size was observed in 260 patients (64.7%), RV free wall and tricuspid annulus motion in 301 (74.9%), RV pressure overload in 297 (73.9%), and RA and central venous pressure in 254 (63.2%) patients. At day 180, the frequency of findings remaining normal or having improved rose slightly to reach 68.7% for RV size, 76.1% for RV free wall and tricuspid annulus motion, 79.8% for RV pressure overload, and 65.9% for RA and central venous pressure. A detailed description of these changes is shown in Table 2. Based on the proposed algorithm, at least one abnormal echocardiographic category was present in 264 (65.7%) patients at baseline, 146 (36.3%) at 6 days and 104 (25.9%) at 6 months.

**Table 2:** Changes of right ventricular function in the study population during follow-up, as assessed by echocardiography

	RV size	RV free wall and tricuspid annulus motion	RV pressure overload	RA and central venous pressure
Day 6 vs baseline				
Persistently abnormal	32 (8.0%)	15 (3.7%)	22 (5.5%)	50 (12.4%)
Deteriorated	15 (3.7%)	7 (1.7%)	6 (1.5%)	21 (5.2%)
Improved	112 (27.9%)	62 (15.4%)	97 (24.1%)	74 (18.4%)
Remained normal	148 (36.8%)	239 (59.5%)	200 (49.8%)	180 (44.8%)
Missing for comparison	95 (23.6%)	79 (19.7%)	77 (19.2%)	77 (19.2%)
Day 180 vs baseline				
Persistently abnormal	15 (3.7%)	9 (2.2%)	3 (0.8%)	40 (10.0%)
Deteriorated	16 (4.0%)	7 (1.7%)	1 (0.3%)	19 (4.7%)
Improved	124 (30.9%)	68 (16.9%)	113 (28.1%)	84 (20.9%)
Remained normal	152 (37.8%)	238 (59.2%)	208 (51.7%)	181 (45.0%)
Missing for comparison	95 (23.6%)	80 (19.9%)	77 (19.2%)	78 (19.4%)

Values represent numbers of patients and the corresponding percentage of the study population. Due to approximation in the first decimal, some percentages do not add up to exactly 100%

*RA* right atrial, *RV* right ventricular

The median value of NT-proBNP was 1448 (406.5–3417) pg/ml at baseline, being almost twice as high (1873 [533–4391] versus 956 [304.3–2468.5] pg/ml) in patients compared to those without RV dilatation; three times as high (2964 [860–5007] versus 1084 [310.3–2662.8] pg/ml) in patients with reduced compared to normal RV free wall and tricuspid annulus motion; more than two-fold increased (2036 [901–4360] versus 955 [303.5–2616] pg/ml) in patients with (versus those without) signs of RV pressure overload; and similarly, also twice as high (1862.5 [700–4537.3] versus 979 [333.8–2664.5] pg/ml) in patients with increased versus normal RA and central venous pressure. Overall, median NT-proBNP levels decreased sharply to 256.5 (94.6–799) pg/ml on day 6, and to 127 (61–280.5) pg/ml on day 180. Nevertheless, the median value of NT-proBNP continued to be relatively high, i.e. 519 (144.8–1463.5) pg/ml in patients with at least one abnormal echocardiographic finding at 6 days; it fell to 148.2 (57.8–405.7) pg/ml in patients with at least one abnormal ultrasound parameter at 6 months.

As shown in Table 3, body-mass index at baseline was associated with the presence of at least one abnormal echocardiographic category 6 days after the acute event, after adjusting for age, sex, prior VTE, history of cancer, history of chronic cardiopulmonary disease, hypotension, hypoxia and tachycardia. Table S3 shows the association of baseline parameters with the presence of at least one abnormal echocardiographic category at 6 months.

**Table 3:** Predictors of abnormal findings of right ventricular function in at least one echocardiographic category at 6 days

Baseline Parameter	Univariable model OR (95% CI)	Multivariable model OR (95% CI)
Age	1.00 (0.98–1.01)	1.00 (0.98–1.02)
Sex (male)	1.07 (0.70–1.64)	1.14 (0.73–1.79)
BMI	1.07 (1.03–1.11)	1.08 (1.04–1.12)
History of VTE	0.72 (0.44–1.16)	0.71 (0.42–1.16)
History of cancer	1.00 (0.54–1.84)	1.07 (0.56–2.04)
History of chronic cardiopulmonary disease	1.67 (1.00–2.80)	1.71 (0.98–2.99)
Systolic BP < 100 mmHg	3.73 (0.79–26.31)	3.58 (0.70–26.79)
Oxygen saturation < 90%	1.49 (0.62–3.58)	1.45 (0.58–3.65)
Heart rate > 100 bpm	0.96 (0.56–1.63)	0.86 (0.49–1.50)
sPESI score	1.26 (0.96–1.67)	–

The analysis was performed in 356 patients without missing values for any of the variables included

*BMI* body-mass index, *BP* blood pressure, *bpm* beats per minute, *CI* confidence interval, *OR* odds ratio, *sPESI* simplified pulmonary embolism severity index, *VTE* venous thromboembolism

## Discussion

The aim of the present analysis was to examine the temporal pattern of changes in RV function, i.e. complete or partial recovery versus persistence or deterioration of abnormal findings, in patients having suffered acute, intermediate-risk PE. We examined the patient population participating in a prospective multicentre multinational single-arm study. Our main results are the following: (i) RV dilatation was the most frequently (41%) reported abnormal echocardiographic finding at the time of the index PE; (ii) in almost two-thirds of the patients, RV function parameters as assessed by echocardiography had already recovered by day 6, and this improvement was maintained over the 6-month follow-up; however, almost one out of four patients had at least one abnormal echocardiographic finding at 6 months; and (iii) the median levels of NT-proBNP remained elevated in patients with at least one abnormal echocardiographic finding at 6 days but decreased and approached normal values at 6 months even among patients with persisting echocardiographic abnormalities.

Our results extend those of previous reports on the temporal changes of RV dimensions and function after acute PE. They may help to improve the level of evidence regarding long-term outcomes since our patient population was included in a prospective management study with standardised initial and chronic (over 6 months) treatment of PE. In an early small observational study dating back to the vitamin K antagonist era, it was reported that RV function normalised in the vast majority of PE patients within the first 5–13 days following treatment initiation [16]. However, another observational cohort study, focusing on 109 patients with submassive PE, demonstrated that an abnormal RV function (indicated by RV dilatation or RV hypokinesia) was still present in 25% of the patients at 6-month follow-up [17]. Furthermore, a substantial proportion (40%) of the patients who were included in the first PEITHO trial and underwent long-term follow-up had one or more indicators of pulmonary hypertension and/or RV dysfunction documented by echocardiography, with no differences between patients randomised to early systemic thrombolysis and those having received placebo [18].

The present study adds to our knowledge on the patients’ long-term course after acute intermediate-risk PE in view of contemporary treatment with a direct oral anticoagulant (in this case, a thrombin inhibitor) over the entire 6-month period, and it also proposes a standardised approach to categorising, analysing and reporting echocardiographic follow-up data. In this regard, it must be emphasised that our ‘algorithm’ was not developed as a new prognostic score in acute PE but rather as a guidance for following RV dysfunction with echocardiographic parameters used in everyday clinical practice. We believe that the parameter groups and the steps developed for our analysis may be useful for future studies with serial assessments of RV (dys)function, especially when multiple centres in different countries are involved. Since the assessment of RV function may be crucial for the resumption of daily activities during follow-up after acute PE [19], an echocardiographic algorithm might also facilitate everyday clinical practice as well as help to harmonise follow-up programs.

Although RV function is expected to recover early after acute PE in the majority of the patients, there are cases where RV recovers at a later stage or even fails to recover completely [19, 20]. This may be due to the presence of pre-existing chronic PE/CTEPH at the time of acute PE and a persistent RV dysfunction already at baseline [21]. The impact of incomplete RV recovery on long-term prognosis, i.e. its correlation with functional limitation, persistent symptoms, poor quality of life and chronic thromboembolic pulmonary disease, with or without pulmonary hypertension, remains to be established.

The observation that obesity was an independent determinant of persistently compromised RV function in our study is of interest but not totally unexpected. Previous studies have reported that obesity negatively affects the cardiovascular system [22] and is a risk factor for RV dysfunction and abnormal RV morphology [23], while also affecting LV size and contractility [24]. An increased body-mass index at baseline has been associated with worse imaging parameters reflecting RV function and higher levels of NT-proBNP, thus underlining the prognostic value of the body-mass index for the outcome of patients with acute PE [25], and justifying the recent call to focus on achieving/maintaining a healthy lifestyle in PE survivors [19, 26].

Strengths of the present study include: (i) the participation of patients from 42 centres across 9 European countries, covering a broad spectrum of clinical settings in which the follow-up took place; (ii) the prospective follow-up of RV function at 6 days and 6 months after the diagnosis of acute PE; (iii) the prospective categorisation of echocardiographic parameters to permit standardised assessment of RV function and its changes over time, and (iv) the assessment of both echocardiographic and biochemical parameters for the evaluation of RV (dys)function and (presumably right) heart failure at baseline and during follow-up. However, our analysis also has some limitations. Firstly, not all parameters related to RV function were available for all patients at all visits. In addition, an association of the kinetics of echocardiographic RV parameters with the primary clinical endpoint of the study, recurrence of symptomatic or fatal VTE at 6 months, could not be established due to the small absolute number of recurrent events [10]. Finally, the algorithm of echocardiographic parameters proposed in the present study needs to be validated in external cohorts and associated with the patients’ clinical long-term prognosis before it can be proposed for broader investigational and clinical use.

In conclusion, our results indicate that in the majority of patients with acute intermediate-risk PE who were switched early (on the third day) to a direct oral anticoagulant, echocardiographic parameters reflecting RV function normalised within 6 days and remain normal throughout the first 6 months. The levels of NT-proBNP also improved during 6-month follow-up. Almost one in four patients, however, still had at least one abnormal echocardiographic finding suggesting some degree of persisting RV dysfunction and possibly the need for continued follow-up over the long term.

### Supplementary Information

Below is the link to the electronic supplementary material.

Supplementary file1 (DOCX 31 KB)
