# Supplementary material for: Recovery of right ventricular function after intermediate-risk pulmonary embolism: results from the multicentre Pulmonary Embolism International Trial (PEITHO)-2
Source: Clin Res Cardiol. 2022 Dec 21;112(10):1372–81. doi: 10.1007/s00392-022-02138-4 (PMC10562278; doi:10.1007/s00392-022-02138-4)
Supplement: Supplementary file 1 — Supplementary file1 (DOCX 31 KB) [file 392_2022_2138_MOESM1_ESM.docx]

**Supplementary Material**

**Table S1 Frequency of echocardiographic findings of right ventricular dysfunction in the study population at baseline, at day 6 and at day 180**

|  | **Baseline** | **Day 6** | **Day 180** |
| --- | --- | --- | --- |
| RV dilatation  Normal RV size  Missing values | 163 (40.6%)  187 (46.5%)  52 (12.9%) | 54 (13.4%)  290 (72.1%)  58 (14.4%) | 33 (8.2%)  310 (77,1%)  59 (14.7%) |
| Reduced RV free wall and tricuspid annulus motion  Normal RV free wall and tricuspid annulus motion  Missing values | 84 (20.9%)  281 (69.9%)  37 (9.2%) | 24 (6.0%)  331 (82.3%)  47 (11.7%) | 17 (4.2%)  333 (82.8%)  52 (12.9%) |
| RV pressure overload  Normal RV pressure  Missing values | 129 (32.1%)  237 (59.0%)  36 (9.0%) | 31 (7.7%)  323 (80.4%)  48 (11.9%) | 4 (1.0%)  348 (86.6%)  50 (12.4%) |
| Increased RA and central venous pressure  Normal RA and central venous pressure  Missing values | 139 (34.6%)  225 (56.0%)  38 (9.5%) | 83 (20.7%)  276 (68.7%)  43 (10.7%) | 66 (16.4%)  287 (71.4%)  49 (12.2%) |

RA = right atrial; RV = right ventricular.

Values represent numbers of patients and the corresponding percentage of the study population.

Due to approximation in the first decimal, some percentages do not add up to exactly 100%.

**Table S2 Baseline characteristics of the patients with and without available echocardiographic data at day 180**

|  | **Patients with echocardiographic follow-up at day 180**  **(n = 353)** | **Patients without echocardiographic follow-up at day 180**  **(n = 49)** |
| --- | --- | --- |
| Women | 169 (47.9%) | 23 (46.9%) |
| Age (years), median (IQR) | 70 (61-77) | 65 (57-79) |
| BMI, median (IQR)  Missing values | 28.3 (25.2-32.0)  4 (1.1%) | 26.4 (24.5-31.2) |
| History of previous VTE | 95 (26.9%) | 12 (24.5%) |
| Major trauma in past 30 days | 12 (3.4%) | 1 (2.0%) |
| Major surgery in past 30 days | 13 (3.7%) | 1 (2.0%) |
| Chronic heart failure | 18 (5.1%) | 6 (12.2%) |
| Chronic pulmonary disease | 39 (11.1%) | 8 (16.3%) |
| Active cancer | 9 (2.6%) | 1 (2.0%) |
| Chronic renal insufficiency | 28 (7.9%) | 2 (4.1%) |
| Systolic BP < 100 mmHg | 7 (2.0%) | 0 |
| Oxygen saturation <90% | 20 (5.7%) | 7 (14.3%) |
| Heart rate ≥ 110 bpm | 33 (9.4%) | 7 (14.3%) |
| NT-proBNP levels (pg/ml), median (IQR)  Missing values | 1501 (425-3417)  106 (30.0%) | 1078 (303-3243)  17 (34.7%) |

BMI = body-mass index; BP = blood pressure; bpm = beats per minute; IQR = interquartile range; NT-proBNP = N-terminal pro brain natriuretic peptide; VTE = venous thromboembolism.

Unless otherwise indicated, values represent numbers of patients and the corresponding percentage of the study population.

**Table S3 Predictors of abnormal findings of right ventricular function in at least one echocardiographic category at 6 months**

| **Baseline Parameter** | **Univariable model**  **OR (95% CI)** | **Multivariable model**  **OR (95% CI)** |
| --- | --- | --- |
| Age | 1.00 (0.99-1.02) | 1.00 (0.99-1.02) |
| Sex (male) | 1.08 (0.68-1.72) | 1.10 (0.69-1.76) |
| BMI (kg/m²) | 1.01 (0.98-1.05) | 1.02 (0.98-1.06) |
| History of VTE | 1.19 (0.71-1.98) | 1.16 (0.69-1.95) |
| History of cancer | 1.13 (0.58-2.11) | 1.08 (0.54-2.06) |
| History of chronic cardiopulmonary disease | 1.17 (0.65-2.05) | 1.16 (0.64-2.05) |

BMI = body-mass index; CI = confidence interval; OR = odds ratio; VTE = venous thromboembolism.

The analysis was performed in 349 patients without missing values for any of the variables included.
